# Rheumatic and Degenerative Mitral Stenosis: From an Iconic Clinical Case to the Literature Review

**DOI:** 10.3390/jcdd11050153

**Published:** 2024-05-17

**Authors:** Francesca Napoli, Ciro Vella, Luca Ferri, Marco B. Ancona, Barbara Bellini, Filippo Russo, Eustachio Agricola, Antonio Esposito, Matteo Montorfano

**Affiliations:** 1 Interventional Cardiology Unit, IRCCS San Raffaele Scientific Institute, 60, Via Olgettina, 20132 Milan, Italy; napoli.francesca@hsr.it (F.N.); vella.ciro@hsr.it (C.V.); ancona.marco@hsr.it (M.B.A.); bellini.barbara@hsr.it (B.B.); russo.filippo@hsr.it (F.R.); montorfano.matteo@hsr.it (M.M.); 2 Cardiovascular Imaging Unit, IRCCS San Raffaele Scientific Institute, 20132 Milan, Italy; agricola.eustachio@hsr.it; 3 School of Medicine, Vita Salute San Raffaele University, 20132 Milan, Italy; esposito.antonio@hsr.it; 4 Clinical and Experimental Radiology Unit, Experimental Imaging Center, IRCCS San Raffaele Scientific Institute, 20132 Milan, Italy

**Keywords:** mitral valve stenosis, TMVR, valve-in-MAC

## Abstract

Mitral stenosis (MS) poses significant challenges in diagnosis and management due to its varied etiologies, such as rheumatic mitral stenosis (RMS) and degenerative mitral stenosis (DMS). While rheumatic fever-induced RMS has declined in prevalence, DMS is rising with aging populations and comorbidities. Starting from a complex clinical case of DMS, the aim of this paper is to review the literature on mitral stenosis by analyzing the available tools and the differences in terms of diagnosis and treatment for rheumatic and degenerative stenosis. Emerging transcatheter techniques, such as transcatheter mitral valve replacement (TMVR) and lithotripsy-facilitated percutaneous mitral commissurotomy (PMC), represent promising alternatives for DMS patients deemed unfit for surgery. In particular, intravascular lithotripsy (IVL) has shown potential in facilitating percutaneous interventions by fracturing calcific deposits and enabling subsequent interventions. However, larger prospective studies are warranted to validate these findings and establish IVL’s role in DMS management. To further enhance this technique, research could focus on investigating the long-term outcomes and durability of mitral lithotripsy, as well as exploring its potential in combination with PMC or TMVR.

## 1. Introduction

### 1.1. A Case of Transcatheter Mitral Valve Lithotripsy without Percutaneous Balloon Valvuloplasty for Palliative Treatment of an Extremely Calcified Degenerative Mitral Valve Stenosis

**Figure 1:**
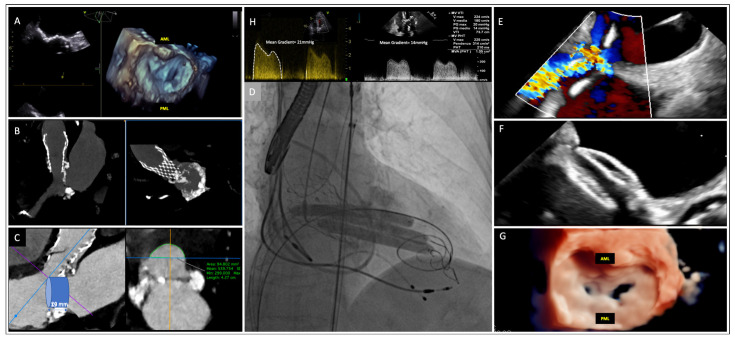
A case of transcatheter mitral valve lithotripsy without percutaneous balloon valvuloplasty for palliative treatment of an extremely calcified degenerative mitral valve stenosis. Panel **A**: TOE showing high calcification extending from the mitral annulus to both the anterior and posterior leaflets, causing severe mitral stenosis. Panels **B**,**C**: Preprocedural assessment CT scans showing the high risk of left ventricle outflow tract obstruction in the case of valve-in-MAC. Panel **D**: Simultaneous inflation of three lithotripsy balloons in the calcified mitral valve. Panels **E**,**F**: Closure of the iatrogenic interatrial septal defect with the Amplatzer ASD Septal Occluder 12 mm (Abbott). Panels **G**,**H**: Final TOE showing trans-mitral gradient reduction from 21 mmHg to 15 mmHg and an improvement in the mitral area from 0.4 to 0.8 cmq.

An 82-year-old woman was referred to our center for severe degenerative mitral stenosis (DMS), which led to NYHA IV symptoms. Transesophageal echocardiography (TOE) revealed a normally functioning CoreValve Evolut R 26 mm (Medtronic, Minneapolis, MN, USA) in the aortic position, a preserved ejection fraction, and severe calcification of the mitral annulus extending to the leaflets, with a severely high mean gradient of 21 mmHg and mild regurgitation (Figure 1A,B,H). The heart team deemed the patient inoperable (EuroSCORE II = 57%), and valve-in-mitral annular calcification (MAC) was excluded because of the high risk of left ventricle outflow tract obstruction (LVOTO) (Figure 1C). Concerns were raised for PMC, both in terms of efficacy (commissures were spared from fusion, Figure 1A) and safety (Wilkin’s score = 16).

Ultrasonic lithotripsy can modify arterial calcium without causing tissue injury [1] and has been recently described to facilitate PMC [2,3]. We speculated that lithotripsy without PMC, delivered through off-label use of commercially available peripheral catheters, could help to disrupt calcium and, thus, facilitate mitral leaflet movement while ensuring safety.

The procedure was carried out under general anesthesia. Due to concerns regarding possible embolization, a TriGuard protection system (Keystone Heart, Tampa, FL, USA) was deployed. After a trans-septal puncture, an Agilis M steerable catheter (Abbott Vascular, Chicago, IL, USA) was advanced into the left atrium, and the mitral valve was crossed with a STORQ wire (Cordis, Hialeah, FL, USA), which was subsequently exchanged for an extra small Safari wire (Boston Scientific, St. Paul, MN, USA). An atrial septostomy was performed with a 14 mm balloon, and three 0.014-inch Grand Slam wires (Abbot Vascular, Abbott Park, IL, USA) were advanced into the left ventricle through an 8 Fr Judkins catheter; the Safari wire was left in place to facilitate the advancement of three 7.0 × 60 mm lithotripsy balloons (Shockwave Medical, Santa Clara, CA, USA), each inserted on a Grand Slam wire. Cardiopulmonary support (CPB) was initiated; thus, the lithotripsy balloons were simultaneously inflated at 4 atm across the valve (Figure 1D—Video S1); four cycles of thirty pulses each were administered during rapid pacing. After termination of CPB, a trans-mitral gradient reduction under 15 mmHg and an improvement of the mitral area from 0.4 to 0.8 cm^2^ were observed (Figure 1G), together with a worsening of regurgitation to a moderate grade. As planned, we preferred not to perform PMC. The left-to-right shunt through the iatrogenic interatrial septal defect (Figure 1E) was closed with an Amplatzer ASD Septal Occluder 12 mm (Abbott, Chicago IL, USA) (Figure 1F). The post-procedural course was uneventful. At the one-month follow-up, a significant clinical improvement was documented: the patient was in a NYHA III class and was able to complete 255 m at the six-minute walking test (6MWT). Echocardiography confirmed a reduced mean trans-mitral gradient (14 mm Hg—Figure 1H) and moderate mitral regurgitation.

### 1.2. Rheumatic and Degenerative Mitral Stenosis

Mitral stenosis (MS) comprises approximately 12% of valvular conditions referred to hospitals [4].

The two main etiologies of MS are rheumatic mitral stenosis (RMS) and degenerative mitral stenosis (DMS). In rare cases (3%), MS may be related to chest radiation, carcinoid heart disease, or inherited metabolic diseases. RMS is the most prevalent form worldwide, especially in developing countries [5]. Improved access to healthcare and widespread use of antibiotics are major factors responsible for the decline in the prevalence of rheumatic heart disease in recent years. DSM is more prevalent in older populations affected by comorbidities such as chronic kidney disease, diabetes, hypertension, and chronic obstructive pulmonary disease. With the rise in life expectancy, the prevalence of DSM is increasing. Recognizing the two different etiologies is an essential first step because of the dramatic differences in pathophysiology, prognosis, disease progression, and interventions.

## 2. Diagnosis

**Figure 2:**
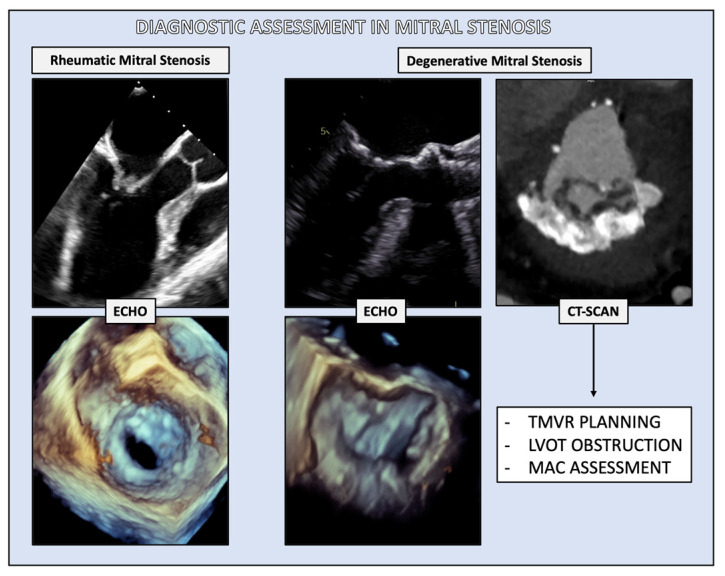
Diagnostic assessment in mitral stenosis. For rheumatic mitral stenosis, both transthoracic and transesophageal echocardiography serve as the primary diagnostic tools; in degenerative mitral stenosis, a CT scan is pivotal, primarily for the pre-procedural planning of a transcatheter procedure, as it could detect pitfalls, such as preventing left ventricle outflow tract obstruction.

The diagnosis of MS typically involves the use of transthoracic echocardiography (TTE) as the primary imaging modality. In certain cases, TTE may be complemented by transesophageal echocardiography (TOE) and computed tomography (CT) for a more comprehensive evaluation, enabling differentiation between RMS and DMS (Figure 2).

Morphological features of the mitral valve (MV), estimation of the mitral valve area (MVA) and mean transmitral pressure gradient (TMPG) are the main components of the echocardiographic assessment of MS.

### 2.1. Morphological Features

RMS could develop as a consequence of rheumatic fever, characterized by inflammatory changes in the valve structures due to a cross-reaction of anti-streptococcal antibodies with the valve tissue [6]. It results in commissural fusion, thickening at the leaflet tips, chordal shortening, and restricted mobility of the posterior mitral valve leaflet. Typically, the rheumatic process involves the mitral leaflet tips, resulting in a funnel-shaped geometry and spared annulus, which are usually the targets of degenerative mitral annulus calcification (MAC).

DMS is the result of chronic non-inflammatory degeneration and subsequent calcification of the fibrous mitral annulus, typically associated with older age, the female gender, and prevalent cardiovascular risk elements like high blood pressure, diabetes, and coronary artery disease (CAD). Calcification of the mitral annulus/valve primarily tends to affect the posterior mitral annulus, manifesting as a calcium deposition between the posterior left ventricular (LV) wall and the posterior MV leaflets [7]. As the disease progresses, anterior mitral annulus involvement and extension of calcification to the myocardium, aortic valve, MV chordae, papillary muscles, and MV leaflets may also occur. Moreover, MAC typically affects the base of the mitral leaflets at the level of the annulus and leads to a tunnel-shaped geometry [8].

When evaluating a potential candidate for percutaneous mitral commissurotomy, the morphological characteristics of the mitral valve serve dual purposes: assessing hemodynamic outcomes and predicting procedural complications. The mitral morphology can be semi-quantitatively described by analyzing the Wilkins score, which is derived from four key morphological features: leaflet mobility, valve thickness, sub-valvular thickening, and valvular calcification. This scoring system assigns grades ranging from 1 to 4 to each feature.

A final Wilkins score below [9] is indicative of a favorable outcome. Generally, optimal hemodynamic outcomes are achieved when the leaflets exhibit thinness, flexibility, and mobility, with commissural fusion proportional to mild calcification and chordal thickening.

### 2.2. Mitral Valve Area (MVA)

Several direct (2D and 3D MV planimetry) and derived echocardiographic techniques (pressure half time, proximal iso-velocity surface area, continuity equation) are commonly used to estimate MVA.

The normal mitral valve orifice is 4 to 6 cm. Symptoms of MS usually occur when the MVA is ≤1.5 cm^2^.

#### 2.2.1. MVA—Planimetry

Planimetry involves directly measuring the MVA in the parasternal short-axis view in the mid-diastole by visually assessing the mitral valve orifice and manually tracing its area [10]. A significant advantage of direct planimetry is its independence from cardiac chamber compliance, flow conditions, or other valvular abnormalities such as mitral regurgitation (MR) or aortic regurgitation (AR), distinguishing it from other echocardiographic methods used to assess MS.

While planimetry at the leaflet tips is deemed reliable for assessing MVA in RMS, its application becomes challenging in patients with MAC because the narrowing typically occurs at the base of the mitral valve at the annular level. Consequently, planimetry at the level of leaflet tips does not accurately represent the true limiting orifice. Additionally, acoustic shadowing caused by calcification of the annulus and leaflets hinders the two-dimensional visualization of the orifice at the base [11]. In this context, 3D echocardiography, due to its ability to demonstrate enface views of the MV structure, has been suggested to overcome these limitations. In summary, 3D echocardiography, especially with TOE, is the most effective method to diagnose and quantify the orifice area in patients with DMS [12].

#### 2.2.2. MVA—Pressure Half Time (PHT)

The calculation of the mitral area through PHT is based on the hemodynamic concept that the rate of reduction of the atrioventricular pressure gradient across the mitral orifice is determined by its cross-sectional area: the smaller the orifice, the slower the gradient reduction. PHT is inversely correlated to MVA, and directly to the left atrial (LA) and left ventricular (LV) compliance and the square root of peak TMPG [13]. In the presence of decreased LV compliance, a phenomenon often observed in the elderly population and commonly associated with DMS, PHT may shorten due to the rapid equilibration of TMPG [8]. This could result in an overestimation of the derived MVA. For these reasons, the measurement of mitral valve area using PHT is reliable in patients with RMS but not in those with DMS.

#### 2.2.3. MVA—Proximal Iso-velocity Surface Area (PISA)

The core principle of the PISA method is that flow acceleration through an orifice generates multiple hemispheric shells with increasing velocity and decreasing radius. Mass conservation dictates that the flow rate at any shell equals the flow across the orifice [10]. The PISA method has been validated for the MVA estimation in RMS [14]. Even though PISA has been shown to be less or not affected by atrioventricular compliance, this measurement is technically challenging in the presence of extensive calcification and there are no validation studies for its use in DMS.

#### 2.2.4. MVA—Continuity Equation

The continuity equation is derived from the principle of mass conservation. It presupposes that the volume passing through the MV and the stroke volume (SV) through the left ventricular outflow tract (LVOT) are equivalent when there are no valvular regurgitant lesions, intracardiac shunting, or irregular heart rhythms [10]. Some studies have demonstrated that the MVA determined by the continuity equation correlates with the values obtained from cardiac catheterization (by Gorlin’s formula) in patients with RMS (correlation coefficient of 0.64) [15]. On the contrary, the continuity equation is not an ideal method for the calculation of MVA in patients with DMS because of the possible effect of measurement errors and the high prevalence of concomitant irregular heart rhythm or valvular regurgitation in this patient population.

### 2.3. Doppler-Derived TMPG

The TMPG is considered a key criterion in assessing MS.

In patients with rheumatic mitral stenosis (RMS), Doppler-derived mean TMPG values show a strong correlation with invasively obtained measurements via trans-septal catheterization [16]. A mean TMPG of 5 mm Hg typically indicates mild MS, while a mean TMPG of 10 mm Hg or higher supports a diagnosis of severe MS [17]. However, there is a lack of similar validation studies for patients with degenerative mitral stenosis (DMS). A study conducted by Hermann et al. [18] utilized an in vitro simulator and mitral valve models, demonstrating a favorable correlation between Doppler-derived and invasively measured TMPG values in the presence of severe mitral annular calcification (MAC). Nonetheless, the Doppler method tended to slightly overestimate the pressure gradient.

### 2.4. The Role of Global Longitudinal Strain

Speckle tracking echocardiography (STE) is a reliable imaging technique that uses the motion of ultrasound backscatter speckles within echocardiographic images to derive myocardial velocities and deformation parameters, providing crucial insights on several cardiac pathological and physiological processes. In particular, the most relevant clinical parameter in STE is the global longitudinal strain (GLS), which reflects the global longitudinal contraction of the myocardium from the base to the apex. The assessment of the extent of left ventricular dysfunction through GLS has both diagnostic and prognostic roles in mitral valve stenosis [19].

Indeed, in MS, myocardial damage can sometimes be more subtle, manifesting primarily as a reduction in global longitudinal strain, especially in the basal and mid-ventricular segments [20]. The underlying reasons are still under investigation, though two hypotheses have been proposed. The first suggests that even after the rheumatic insult has subsided, inflammation may persist, leading to fibrosis and scarring of the mitral apparatus, extending to the endocardium and myocardium [21]. Another hypothesis points out that the fibrotic mitral valve may induce posterobasal wall motion abnormalities due to a tethering effect [22].

Finally, Segupta et al. demonstrated that strain improves post-valvuloplasty. One possible explanation is that valvulotomy increases preload, thereby augmenting left ventricular end-diastolic volume (LVED). This increase in LVED is believed to subsequently enhance left ventricular function [23].

### 2.5. Evaluation of MAC

Between 9% and 15% of the general population may have MAC without hemodynamic significance, as calcified nodules are typically located at the base of the leaflets and associated with valve leaflets thickening, but without significant restriction of leaflet movement and/or obstruction to flow [24,25]. Although MAC is most commonly an asymptomatic and incidental finding, it may also be associated with mitral valve stenosis or mitral regurgitation.

At TTE evaluation, MAC typically manifests as a distinct, irregular echo density with a shelf-like appearance, often accompanied by acoustic shadowing. Moreover, there is no standardized echocardiographic definition or grading criteria for MAC severity. In epidemiological studies, the severity of MAC has been evaluated based on either the maximum thickness of the echo density (with a threshold of 4.4 mm indicating severe MAC) or the extent of mitral annular involvement observed in the parasternal short-axis view. Focal calcification denotes mild MAC, while extensive calcification affecting more than half of the annular circumference or encroaching into the left ventricular inflow tract signifies severe MAC [26].

The TTE evaluation of MAC has several limitations, such as the differentiation of calcium from dense collagen and the quantification of MAC solely based on subjective visual scoring. To overcome these limits, an electrocardiography-gated contrast-enhanced CT scan is useful. The Agatston score offers an alternative method for assessing the severity and distribution of MAC, although standardized cutoff values for grading are lacking [27].

Additionally, a CT scan facilitates reproducible estimations of MVA through planimetry. MVA derived via CT planimetry demonstrates a strong correlation with values obtained through cardiac catheterization (Gorlin’s formula). Research by Lembcke et al. proposed a CT-determined MVA of 1.7 cm^2^ as the optimal cutoff to differentiate mild from moderate-to-severe mitral stenosis (MS) [28].

The advanced imaging capabilities make the CT scan an indispensable tool for planning potential interventions or surgeries for DMS [29]. Within this context, CT enables the simulation of MV replacement with various prosthetic sizes, allowing for precise prediction of neo-anatomy and potential complications such as LVOT obstruction.

## 3. Treatment Options

### 3.1. Rheumatic Mitral Valve Stenosis

Therapeutic options for patients affected by severe RMS include percutaneous mitral commissurotomy (PMC) and MV surgery. Medical therapy is limited to addressing complications such as atrial fibrillation (AF) and congestive heart failure (CHF), as well as implementing secondary prophylaxis to prevent the recurrence of acute rheumatic fever (ARF) [30].

The type of treatment, as well as the timing, should be decided based on clinical and anatomical characteristics, considering contraindications and unfavorable characteristics for PMC.

Contraindications for PMC are MVA > 1.5 cm^2^, left atrial thrombus, more than mild regurgitation, severe calcification, the absence of commissural fusion, and severe concomitant other valve disease or CD.

Unfavorable characteristics for PMC can be defined by the presence of several of the following characteristics.

- Clinical characteristics: old age, history of commissurotomy, New York Heart Association class IV, permanent AF, severe pulmonary hypertension.
- Anatomical characteristics: echocardiographic Wilkins score >8, Cormier score 3 (calcification of mitral valve of any extent as assessed by fluoroscopy).

In this context, and according to ESC guidelines [31], PMC or MV surgery is indicated in the following cases:

- PMC is recommended for symptomatic patients without unfavorable characteristics. On the contrary, MV surgery is recommended in symptomatic patients who are not suitable for PMC due to the presence of unfavorable characteristics. Finally, in symptomatic patients with suboptimal anatomy but not unfavorable characteristics, PMC should be considered.
- PMC should be considered in asymptomatic patients without unfavorable clinical and anatomical characteristics, high thromboembolic risk, or high hemodynamic decompensation, such as systolic pulmonary pressure > 50 mmHg at rest.

Approximately 80% of symptomatic individuals with severe RMS are eligible for PMC, with only 20% remaining for surgery due to unfavorable anatomical conditions and an elevated Wilkins score [9].

Furthermore, research indicates that the long-term results are similar between PMC and open mitral commissurotomy [32].

When surgery is pursued, the dilemma of choosing between repair and replacement procedures arises. This issue is very pertinent for young patients hailing from underprivileged regions worldwide, who encounter obstacles in adhering to any medical regimen, including anticoagulation therapy. In such instances, the significance of repair is undeniably crucial. On the other side, it should be considered that the durability of repair of the rheumatic mitral valve is generally poorer than in a non-rheumatic valve [33], and the risk of needing reoperation given limited resources. When mitral valve repair is not feasible, the dilemma of choosing between mechanical prostheses and bioprostheses exists. Nonadherence to anticoagulation therapy remains a significant consideration against a mechanical valve, while the rapid progression of bioprosthetic degeneration is the primary limitation.

### 3.2. Degenerative Mitral Valve Stenosis

Patients affected by DMS are generally elderly with multiple comorbidities and are often high-risk candidates for surgery with anatomical features that are not suitable for PMC. Thus, contrary to RMS, there is a consensus that medical therapy is the first-line approach for patients with DMS.

**Figure 3:**
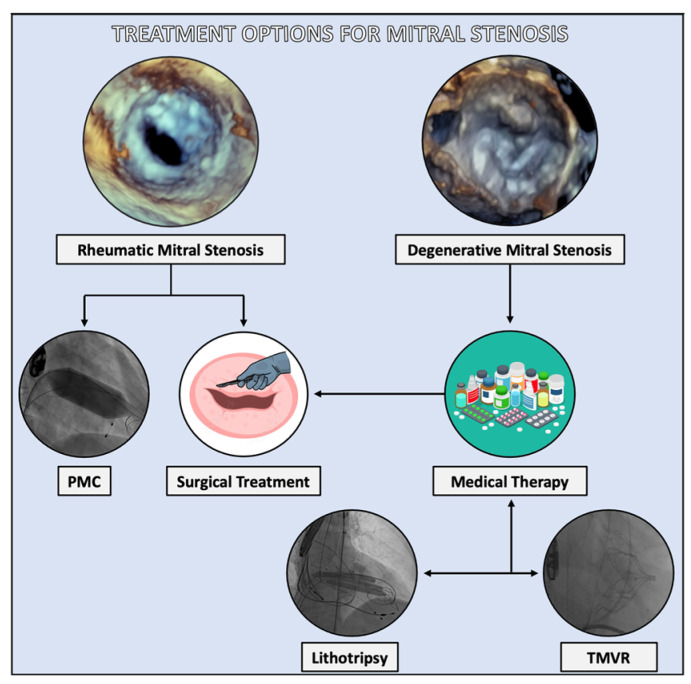
Treatment options for mitral stenosis. Therapeutic options for patients affected by severe rheumatic mitral stenosis include percutaneous mitral commissurotomy (PMC) and mitral valve surgery, with a limited role in medical therapy. Conversely, patients affected by degenerative mitral stenosis (DMS), who are generally elderly with multiple comorbidities, are often high-risk candidates for surgery and with anatomical features not suitable for PMC. Thus, medical therapy is the first-line approach in DMS. However, in symptomatic patients despite optimized therapy, the surgical option exists. Emerging transcatheter solution, with lithotripsy to reduce the stenosis severity or facilitate percutaneous valve replacement could be considered in selected cases.

Intervention, whether surgical or transcatheter, is reserved for highly symptomatic patients refractory to diuresis and heart rate control (Figure 3).

#### 3.2.1. Medical Therapy

Patients with severe mitral stenosis have poor functional reserve, and the mean TMPG easily increases with tachycardia or high flow. In this context, heart rate control with beta-blockers, calcium-channel blockers, or ivabradine for patients with sinus rhythm can lengthen diastole and, thereby, improve LV filling.

Moreover, because of the high thromboembolic risk, anticoagulation with vitamin K antagonists (VKA) is recommended in patients with concomitant atrial fibrillation and those in sinus rhythm with a history of systemic embolism and/or evidence of a thrombus in the left atrium.

Treatment of comorbidities may have some effect on delaying the progression of MAC, but the level of evidence is weak. For example, in patients with chronic kidney disease (CKD), the use of phosphate binders, vitamin D receptor agonists, calcimimetics, and sodium thiosulfate might also slow the progression of valve calcification. Similarly, due to the association between MAC and atherosclerosis, statins may lower the calcium burden [34].

Surgical intervention should be delayed until symptoms are severely limiting and cannot be managed by medical therapy, considering that MV surgery presents additional challenges in these patients due to extensive calcification. Therefore, it is imperative to devise an alternative percutaneous treatment approach for individuals with DMS who are unfit for surgery or at high risk for it.

#### 3.2.2. Surgical Treatment

Historically, surgical mitral valve replacement (MVR) has been the preferred approach for managing symptomatic DMS patients.

Calcification of the mitral annulus, whether extending to the leaflets or not, introduces a range of technical hurdles in valve repair or replacement procedures. Indeed, the presence of a posterior annular calcium bar inhibits the reconstruction, reduction, and realignment of the mitral annulus.

Additionally, surgical replacement depends on the integrity of the fibrous mitral annulus to serve as a stable anchor for the valve sutures. At the same time, debridement of calcified tissue carries high risks, as it could lead to atrioventricular separation or harm to the circumflex coronary artery.

Spencer et al. reported 14 cases of LV rupture associated with posterior MAC in 4 cases and annular debridement in 3 cases [35]. Moreover, another potential complication reported by MacVaugh [36] is intractable hemorrhage from the ventricular wall. In addition to these catastrophic complications, a paravalvular leak represents another significant limitation of valve replacement in cases of severely calcified annuli.

In these instances, where there is no consensus strategy for the treatment of MAC, the cavitron ultrasonic surgical aspirator (CUSA) is a useful adjunct technique in surgical management, while open-surgery TMVR emerges as a promising alternative.

- The CUSA operates as an ultrasonic device, targeting tissue destruction, followed by washing the area and aspirating the fragmented mass. It enables the remodeling of the annulus, simplifies suture placement, and facilitates the seating of the prosthetic valve. However, it is important to note that, while clinical cases have demonstrated its efficacy, there is a lack of scientific studies specifically evaluating its use in this context [37].
- Open-surgery transcatheter mitral valve replacement (TMVR) stands out as a groundbreaking advancement in cardiac surgery. Indeed, while percutaneous TMVR has shown encouraging results, it faces limitations such as para-valvular leaks and prosthesis migration (as illustrated in the following sections). In contrast, open surgery TMVR offers a solution by allowing direct access to address these issues. Through precise suturing of atrial tissue onto the transcatheter valve prosthesis skirt, para-valvular leaks can be effectively resolved. Additionally, annular prosthesis mismatch, a common challenge in percutaneous TMVR, can be overcome by implanting a complete annuloplasty ring during valve-in-ring implantation. Moreover, the risk of left ventricular outflow tract obstruction (LVOT) associated with transcatheter procedures can be mitigated through techniques like excision of the anterior mitral leaflet and orientating the prosthesis away from the LVOT during open surgery [38].

#### 3.2.3. Percutaneous Mitral Commissurotomy (PMC)

In patients with non-rheumatic calcific MS, leaflets are usually affected with no commissural fusion, and the role of PMC is quite limited. Indeed, in ESC Guidelines, an IIa/C recommendation is given to the percutaneous approach in significant symptomatic MS (irrespective of the etiology) without unfavorable characteristics for the PMC [31]. In cases of heavily calcified mitral valves (rheumatic or degenerative), “uncontrolled balloon-based dilatation” could lead to tears or ruptures of the calcific tissue in the leaflets (not splitting of the commissures) and severe acute MR that requires emergent surgery. The frequency of this complication ranges from 2% to 19% [39].

In this context, two novel procedures have emerged that may challenge this paradigm in native valves: the transcatheter mitral valve replacement (TMVR) and the lithotripsy-facilitated transcatheter mitral valve procedure.

#### 3.2.4. Transcatheter Mitral Valve Replacement

TMVR is emerging as a promising alternative to surgical techniques.

In patients with MAC and MS, balloon-expandable transcatheter valves (BEVs) designed for the aortic valve can be implanted with MAC acting as an anchor. Dedicated TMVR devices such as Tendyne TM (Abbott, USA) and Intrepid TM (Medtronic, USA) valves are currently under assessment in MAC patients, with initial findings from the MAC subgroup already documented [40,41,42,43].

BEVs designed for the aortic valve can be delivered via a trans-atrial, trans-septal, or trans-apical approach. It is important to note that the direction in which the valve is mounted on the balloon (if transseptal) must be opposed to the one for TAVR.

Five observational studies have been published since 2017, exploring TMVR with a variety of aortic BEVs (Edwards SAPIEN valve, Lotus, Direct Flow) in prohibitive surgical risk patients. Mortality at the one-year follow-up is around 50% and the most frequent adverse event is the obstruction of the LVOT. When evaluating the three different access routes for TMVR, the trans-atrial approach emerges as the most promising with less one-year mortality rates of 35% compared to those of the transapical (57%) and transseptal (63%) approaches, along with superior technical success rates (89% compared to 71% and 65%, respectively) [44,45,46,47,48].

Guerrero et al. also spearheaded the mitral implantation of transcatheter valves trial (MITRAL trial) and disclosed its 1-year findings. This prospective investigation explored the viability of TMVR using BEVs (SAPIEN XT). The study enrolled 31 participants, with technical success observed in 74.2% of cases. Among them, three patients experienced LVOT obstruction. All-cause mortality rates at 30 days and 1 year were recorded at 16.7% and 34.5%, respectively [49].

The most recent 2-year follow-up results from the MITRAL trial represent the longest follow-up period available thus far. At 2-years, mortality was observed in 39.3% of patients with TMVR. During the period between the 1-year and 2-year follow-up, one non-cardiovascular death was recorded, and there were no instances of hospitalizations due to heart failure. Extended-term data regarding TMVR in MAC patients have not yet been made accessible [50].

The main limitations of TMVR in MAC are as follows:

- Paravalvular leakage (PVL) occurs because the mitral annulus is not circular and very calcific. Assessment of PVL is crucial as it could be linked to hemolysis or hemodynamic complications. The existence of intra-procedural PVL might necessitate valve dilatation, while persistent significant PVL might call for transcatheter closure of the PVL. This complication could be avoided by using an oversized prosthesis [51].
- Bioprosthesis embolization, especially in cases of non-complete circumferential MAC. Guerrero et al. introduced a computed tomography (CT)-derived MAC scoring system to assess the severity of MAC and predict valve embolization during TMVR using balloon-expandable aortic transcatheter heart valves. This scoring system considers average calcium thickness (mm)—degree of the annulus circumference involved—calcification at one or both fibrous trigones—calcification of one or both leaflets. Mild-to-moderate MAC presents a notably elevated probability of valve embolization, while severe MAC entails a diminished likelihood of valve embolization or migration [52].
- The impossibility of implanting any available prosthesis in a very large mitral annulus.
- Thrombus formation due to a high turbulence rate, low cardiac output, and the resultant slow movement of leaflets. Anticoagulation for at least 6 months may limit this complication [40].
- Atrio-ventricular groove injury especially in small left ventricles, severe MAC, and oversized TMVR devices [53].
- Durability in TMVR is less than in TAVR probably due to prosthesis deformation. Furthermore, MV is a dynamic and intricate entity, where the interplay among its components can influence the durability and functionality of devices, even under optimal implantation conditions [53].
- Residual inter-atrial septal defect: once the procedure is finished, an atrial septal defect closure may be needed in 30–50% of the patients to avoid left-to-right shunts.
- Embolic stroke that can be limited by cerebrovascular protection devices.
- Left ventricular outflow tract (LVOT) obstruction due to the permanent displacement of the anterior mitral leaflet towards the interventricular septum, creating a narrow and elongated neo-LVOT. This obstruction remains fixed. The obstruction becomes dynamic when neo-LVOT induces Bernoulli forces, pulling the mitral leaflet against the interventricular septum during systole. The strongest CT-predictors of LVOT obstruction are the LVOT area and the predicted neo-LVOT area (after valve implantation). By simulating the placement of a virtual valve in the mitral annulus, pre-planning CT enables the prediction of the neo-LVOT area across different systolic phases of the cardiac cycle. Yoon et al. showed that an estimated neo-LVOT area of ≤1.7 cm^2^ predicted LVOT obstruction with a sensitivity and specificity of 96.2% and 92.3%, respectively. In addition, aorto-mitral angulation close to 90°, a small left ventricular cavity, and basal hypertrophy (<15 mm) were also found to be risk factors for post-TMVR LVOT obstruction [54]. In these cases, pre-emptive techniques to avoid LVOT obstruction, such as LAMPOON (laceration of the anterior mitral leaflet to prevent outflow obstruction) or ASA (alcohol septal ablation) technique should be performed. The LAMPOON technique consists of intentional laceration of the anterior MV leaflet using catheters placed in the left atrium and LV to puncture the anterior leaflet and lacerate it with electrocautery. Early experience with this technique on 30 patients achieved midline laceration of the anterior leaflet in 100% of patients and a 30-day survival rate of 93%. This technique is being studied in a prospective single-arm trial [55].

An alternative strategy is ASA, which is used as a bail-out option for patients with LVOT obstruction from a thick basal septum during TMVR. In patients at high risk of LVOT obstruction, as predicted by preprocedural planning CT, 30 patients underwent pre-emptive ASA, which resulted in a median increase in the neo-LVOT area, and overall mortality at 30 days was 10% [56].

An alternative investigational approach in patients where anterior laceration of the MV leaflet is not feasible or coronary anatomy is not favorable for ASA is percutaneous cardiac ablation of a thickened basal septum with 3D electroanatomic mapping and guidance to avoid ablation of conduction tissue [57].

## 4. Physical Principles and Potential Use of Lithotripsy

The intravascular lithotripsy (IVL) device (Shockwave Medical, Santa Clara, CA, USA) converts electrical energy into mechanical energy while inflating a low-pressure balloon [58]. Mechanical energy consists of sonic waves, which propagate from a balloon-based catheter to the nearby tissue, aiming to selectively fracture both superficial and deep calcium deposits while minimizing damage to soft tissue. Unlike other debulking techniques, the calcium fragments produced by IVL therapy remain in place, lowering the risk of distal embolization [1].

The coronary IVL system includes a portable, rechargeable power source, a connector cable equipped with a push button for manually regulating the delivery of electric pulses, and a 6 Fr compatible, rapid-exchange, semi-flexible balloon catheter for utilization over a standard angioplasty 0.014 in. guidewire. The balloons range in diameter from 2.5 to 4.0 mm, with a standard length of 12 mm, and integrate two radiopaque lithotripsy emitters 6 mm apart and two conventional markers at the proximal and distal edges of the balloon. These emitters receive electrical pulses from the generator, causing the fluid within the balloon to vaporize and generate rapidly expanding and collapsing bubbles. These bubbles transmit unfocused circumferential pulsatile mechanical energy into the vessel wall, manifesting as sonic pressure waves approximately equivalent to 50 atm. IVL therapy comprises a sequence of 10 pulses, constituting one cycle, delivered in 10 s.

IVL, initially developed for the treatment of kidney stones, has been further validated as a debulking system for calcific lesions in peripheral and coronary vessels [59]. In recent years, it has emerged as a facilitation system for patients with calcified structural heart disease requiring valve treatment.

## 5. Lithotripsy-Facilitated PMC

Interventional cardiologists have been confronted with the dual challenge presented by the inability to perform PMC in severely calcified DMS cases on one hand, and the increasing cardiovascular risk factors and advancing age contributing to a growing number of patients afflicted with DMS on the other. This, together with current clinical practice and real-world scenarios, has, therefore, led to the expansion of the original technique in selected DMS, giving rise to the lithotripsy-facilitated PMC.

The procedure of applying shockwave pulses to MV is demanding due to the requirement of general anesthesia, transesophageal echo guidance, two femoral venous accesses (one for transeptal puncture and the other for pacing), and arterial access for blood pressure monitoring.

After obtaining access and with TOE guidance, the transeptal puncture is performed in the inferior and posterior position of the fossa ovalis. Then, advance a high-support wire into the LV ventricular with the help of a guiding catheter (such as multipurpose) and create an interatrial septostomy with a 12 mm balloon. This is mandatory due to the size of the devices and sheaths to be advanced to the mitral valve. The next step consists of crossing the mitral valve with two or three long high-support 0.014-inch wires and then advancing two or three lithotripsy balloons (Shockwave Medical) across the MV. Thereafter, during rapid pacing (120 bpm), simultaneous inflations of all balloons will deliver a total of 90 pulses from each balloon. Finally, interventional cardiologists complete the PMC with large balloons appropriately sized to the mitral valve area and diameters over a Safari wire.

According to our knowledge and the current state of the art, data on the utility of IVL in facilitating PMC have only been described in case reports or case series (see Table 1).

- In 2019, Eng et al. published the first-in-human case of IVL-facilitating PMC in DMS. An 81-year-old severely symptomatic for DMS with a mean TMPG of 11 mmHg, was excluded from surgery due to the extremely high risk and was deemed not suitable for TVMR due to a large MVA (810 mm^2^). There was concern for the efficacy of PMC due to the elevated calcium burden. In this context, lithotripsy-facilitated PMC was performed; two 7 mm and one 6.5 mm lithotripsy balloons were simultaneously inflated across MV; the TMPG did not change. Subsequent PMC with a 28 mm balloon lowered the mean TMPG to 2 mmHg [2].
- In 2020, Sharma et al. published the first-in-human case of IVL-facilitating PMC in calcific rheumatic MS. An 86-year-old male affected by calcific RMS with TMGP of 14 mmHg, underwent off-label use of IVL before. Three 7.0 × 6.0 mm lithotripsy balloons were simultaneously inflated across the mitral valve. Transesophageal echocardiography showed a reduction in the mean TMPG to 6 mmHg. PMC was subsequently performed with 24 mm and the final TMPG was 4 mmHg. Unlike the findings of Eng et al., this case demonstrated a decrease in the average transmitral pressure gradient following IVL alone. This variation could be attributed to differences in the etiology of MS (calcific-RMS versus DMS), where IVL rendered calcified leaflets more flexible, thus alleviating stenosis. Moreover, smaller MVA and the deployment of three 7 mm balloons potentially facilitated improved tissue contact and lithotripsy energy delivery in our case [3].
- Kassar et al. described the case of an 84-year-old man affected by DMS with a TMPG of 8 and 3D MVA of 0.6 cm^2^. He had an extremely high surgical risk and was excluded from PMC due to his high Wilkins score and from TMVR due to his extremely small MVA. It was the first case of IVL-facilitated PMC successfully performed (post-procedure Gorlin-MVA 3.1 cm^2^—3D TOE MVA 1.5—TMPG 4) with new 8-mm Shockwave balloons (compared with 7-mm balloons) that have the capability of delivering 2 shocks per second, aiding in reducing the occlusion time of the mitral valve. The length of these balloons ensured stability, eliminating the need for pacing [60].
- Sant-Ruiz et al. described the first case of lithotripsy-facilitated PMC performed in a rheumatic setting at the same time as TAVR in a “one-step approach”. An 87-year-old affected by MS, severe aortic stenosis, and end-stage CKD, has undergone IVL-facilitated PMC and subsequent transcatheter aortic valve implantation (TAVI), definitely proving the safety and efficacy of this procedure even when combined with aortic valve interventions [61].
- Chadda et al. described a case of a 69-year-old patient undergoing IVL-facilitated PMC, emphasizing the importance of a large lumen transseptal support catheter to aid the deployment of the IVL balloons rather than wrestling with three bare wire systems through the interatrial septum. Indeed, the operators obtained sufficient support to perform simultaneous hugging double balloon inflations. Nonetheless, for upcoming cases, they advise contemplating the utilization of a 16 F Abbott Fast-Cath (Abbott Vascular) or a 16 F Cook Medical Mullins sheath (Cook Medical, Bloomington, IN, USA) if the valve orifice necessitates the simultaneous positioning of three 7 mm IVL balloons. In such instances, the higher risk of stroke can be alleviated through the application of a cerebral protection system [62].
- Giustino et al. recently published the largest case series to date, focusing on the intrahospital outcomes of 24 consecutive patients with severe DMS who underwent IVL-PMC. All patients were excluded from surgery. The mean age of the patients was 77 and most of them were in NYHA functional class III or IV. A cerebral protection device (Sentinel, Boston Scientific) was used in 70.8% of cases, and stroke was observed in only one case in which a Sentinel device was not used. A subsequent PMC was performed in 21 cases. Regarding the mean TMPG after the procedure, the absolute difference from baseline was −5 mmHg, one patient had a residual mean TMPG > 10, and 7 patients > 5 mmHg. Complications observed were right ventricular perforation (4.2%), pericardial effusion (4.2%), and pacemaker implantation due to advanced atrioventricular block developed after ASA (8.4%) [63].

Based on case reports and case series, IVL-facilitated PMC in patients with severe MAC appears feasible and safe, and results in a substantial reduction in MV gradients. Larger prospective studies in this high-risk population are required to validate these findings.

## 6. Lithotripsy-Facilitated TMVR

As previously mentioned, TMVR for native valve disease is a viable option in high-surgical-risk patients. However, managing MAC remains very challenging for both BEVs, due to the high risks of annulus injury and for SEF because of constraints during deployment and subsequent pinwheeling of the valve leaflets with residual MR.

In this context, the utilization of IVL to treat calcified mitral annulus and leaflets prior to TMVR could enhance leaflet flexibility, improve annular compliance, reduce fibro-elastic recoil, and prevent valve frame deformation.

Seshiah et al. recently published the first-in-human case of IVL-assisted transeptal TMVR with the Intrepid valve (Medtronic) in an 83-year-old patient with severe calcific-RMS and MR. At TTE evaluation, the mean TMPG was 10 mm Hg, the mitral valve area by continuity equation was 0.9 cm^2^, and MR was grade 3. Due to the extremely high surgical risk, he was scheduled for IVL-assisted TMVR. Through the right femoral vein and transeptal puncture, two 8 × 60 mm shockwave balloons were advanced across the mitral valve annulus, and both inflated. Thereafter, MV pre-dilatation with a 26-mm True balloon was performed and a 48-mm Intrepid valve was advanced and implanted. Thereafter, due to evidence of moderate MR and moderate valve frame deformation, valve post-dilatation with a 28-mm Z-MED balloon was performed [60].

Pre-discharge TTE and at 30 days revealed mild MR, a mean TMPG of 4 mmHg, and progressive circularization of the valve frame.

Although anecdotal, this case shows that IVL is effective in modifying MAC, as demonstrated by an immediate increase in valve dimension (after IVL) and progressive circularization/expansion of the valve over time.

## 7. Lithotripsy-Facilitated Transcatheter Edge-to-Edge Mitral Valve Repair

MAC is also prevalent in patients with MR, and it is associated with high morbidity, mortality, and worse cardiac surgical outcomes. In this setting, a need for less invasive and yet efficient alternative therapies arises.

Transcatheter edge-to-edge mitral valve repair (TEER) with the MitraClip system (Abbott Vascular) has been established as a valid alternative to surgery in high-surgical-risk patients with severe MR. However, its efficacy in patients affected by MAC remains uncertain due to more complex anatomies (increased subvalvular apparatus calcification and more eccentric jets), higher MR grades, and a higher mean TMPG.

Recent findings show that MAC is not a contraindication to TEER, in the absence of significant mitral stenosis or leaflet calcification. Even though these patients show a higher residual TMPG, it is not independently associated with an increase in all-cause, cardiovascular mortality, or unplanned cardiac readmissions at follow-up. Moreover, there were no cases of clip detachment, embolization, or stroke [65].

To reduce post-implantation residual TMPG due to MAC, IVL may be useful.

Our experience is limited to a single case report published by Neil et al. A 71-year-old man noted for severe calcific MR with a valve area of 2.2 cm^2^ and a mean TMPG of 5 mmHg, was banned for surgery and deemed unsuitable for TMVR due to the high risk of LVOT obstruction. Thus, he was scheduled for IVL-facilitated TEER. At first, IVL with two 7 × 60 mm balloons across the mitral valve was performed with minimal reduction of TMPG (4 mmHg). Secondly, mitral valvuloplasty with 30 and 33 mm NuMED balloons was done.

This facilitated the successful implantation of a MitraClip NTW, with a final mild MR and a mean gradient of 4 mmHg [64].

## 8. Future Directions

Dealing with DMS presents an escalating challenge, particularly with calcific deposits emerging as a formidable enemy for interventional cardiologists. Unfortunately, as global populations continue to age progressively, an increasing number of patients will require calcium-modifying technologies in percutaneous valvular interventions.

In the context of lithotripsy-facilitated PMC, promising outcomes have been evidenced with case reports and series showing significant reductions in mean TMPG and augmentation of MVA. To further enhance this technique:

- Research could focus on investigating the long-term outcomes and durability of mitral lithotripsy, as well as exploring its potential in combination with PMC or TMVR. In this setting, the creation of an IVL-assisted PMC registry would be beneficial.
- Advancements in lithotripsy may involve improving procedural techniques for better safety and effectiveness. In this scenario, the development of an IVL transcatheter balloon specifically for MV application may be useful.
- It is crucial to optimize patient selection criteria and develop tailored treatment approaches based on individual anatomical and clinical characteristics. In this context, investigating the patterns and severity of MAC would be beneficial, assuming a varied response to IVL depending on MAC features.

With respect to TMVR for MS, it has emerged as a viable option for patients at high surgical risk. However, refinement is necessary to optimize this technique. For example, refining anatomical definition through comprehensive pre-procedural planning with CT is crucial in avoiding complications such as LVOT obstruction, valve migration, embolization, and paravalvular mitral regurgitation.

Thereafter, we are waiting for the results of two trials exploring the clinical outcomes of balloon-expandable transcatheter valves intended for the aortic position but deployed in the mitral valve. The MITRAL II pivotal trial (mitral implantation of transcatheter valves—NTC04408430) compares the difference in clinical outcomes between patients undergoing SAPIEN 3 or SAPIEN 3 ultra valve implantation and the control arm (medically treated). Moreover, the SITRAL trial (surgical implantation of the transcatheter valve in native mitral annular calcification—NTC02830204) investigated the clinical outcome of 30 patients treated with surgical SAPIEN 3 valve implantations.

There are currently two ongoing trials assessing the use of valves specifically designed for the mitral position: the Tendyne transcatheter bioprosthetic valve (Abbott Structural, Santa Clara, CA, USA) and the Intrepid valve (Medtronic, Minneapolis, MN, USA). Although both trials focus on the treatment of MR rather than MS, they will be suitable for the treatment of MS in the presence of MAC.

Finally, further studies are needed to confirm the efficacy of IVL in pre-TMVR implantation to enhance leaflet flexibility, improve annular compliance, reduce fibro-elastic recoil, and prevent valve frame deformation.

## Figures and Tables

**Table 1:** Summary of the main evidence about IVL treatment in degenerative mitral stenosis.

IVL Facilitated PMC	Design	Patient Characteristics	Procedural Details	Outcome	Innovations
- Eng et al. (2019) [2]	case report	81 years old, severe symptomatic DMS mean TMPG 11 mmHg MVA 810 mm^2^ (not suitble for TMVR)	2 IVL balloons (7.0 mm + 6.5 mm) + 28 mm PMC balloon	Lowered mean TMPG to 2 mmHg	First-in-human **IVL** facilitating PMC in **DMS**
- Sharma et al. (2020) [3]	case report	86 years old, severe simptomatic calcific RMS: mean TMPG 14 mmHg	3 IVL balloons (7.0 × 6.0 mm) + 24 mm balloon	Lowered mean TMPG to 4 mmHg	First in human **IVL** facilitating PMC in **calcific RMS**
- Kassar et al. (2022) [60]	case report	84 years old, severe symptomatic DMS mean TMPG 8 mmHg Wilkins score = 8 (not suitble for PMC) MVA 60 mm^2^ (not suitble for TMVR)	2 IVL balloons (8.0 × 60 mm) + 25 mm PMC balloon	Lowered mean TMPG to 4 mmHg	First case of **8 mm IVL ballon** delivering 2 shock/second aiding in reducing MV occlusion time
- Sant Ruiz et al. (2021) [61]	case report	87 years old, severe DMS + severe aortic stenosis TMPG 17 mmHg mean transortic DP 29 mmHg	3 IVL balloons (7.0 × 60 mm) + 26 mm PMC-balloon + TAVR (23 mm Acurate Neo 2)	Lowered mean TMPG to 4 mmHg Lowered mean aortic PG to 8 mmHg	First case of **IVL** facilitating PMC + **TAVR** in »one step approach»
- Chadda et al. (2021) [62]	case report	69 years old, severe DMS mean TMPG 10.8 mmHg Wilkins score = 9 (not suitble for PMC) MVA 86 cm^2^ (not suitble for TMVR)	3 IVL balloons (7.0 × 40 mm) + 24 mm PMC-balloon	Lowered TMPG 3 mmHg	First case emphatising large lumen transseptal support catheter
- Giustino et al. (2024) [63]	case series	24 patients, severe DMS	70.8% use of cerebral protection 87.5% IVL facilitated PMC 12.5% successful IVL alone **Complications:** - RV perforation (4.2%) - pericardial effusion (4.2%) - PM i (8.4%)	mean TMPG absolute difference from baseline was −5 mmHg	First-in-human successful **IVL without PMC** performed
**IVL facilitated TMVR**					
- Seshiah et al. (2022) [60]	case report	83 years old, severe calcific-RMS + MR TMPG 10 mmHg MVA 90 mm^2^ MR grade 3	2 IVL balloons (8.0 × 60 mm) + TMVR (Intrepid 48 mm)	mean TMPG 4 mmHg	First-in-human case of **IVL** assisted transeptal **TMVR**
**IVL facilitated TEER**					
- Fam NP et al (2022) [64]	case report	71 years old, severe calcific MR + MAC mean TMPG 5 mmHg MVA 2.2 cm^2^ Not suitble for TMVR due to high risk LVOT	2 IVL balloons (7.0 × 60 mm) + MV valvuloplasty (30–33 mm NuMED ballons) + MitraClip NTW implantation	Mild MR mean TMPG 4 mmHg	First-in-human **IVL** facilitated **Mitraclip**

## Supplementary Materials

The following supporting information can be downloaded at https://www.mdpi.com/article/10.3390/jcdd11050153/s1, Video S1: Simultaneous inflation of three lithotripsy balloons in a calcified mitral valve resulted in improved leaflet opening without significant worsening of mitral regurgitation.

## Abbreviations

Degenerative mitral stenosis	(DMS)
Mitral annular calcification	(MAC)
Left ventricle outflow tract obstruction	(LVOTO)
Cardiopulmonary support	(CPB)
Mitral stenosis	(MS)
Rheumatic mitral stenosis	(RMS)
Degenerative mitral stenosis	(DMS)
Transthoracic echocardiography	(TTE)
Transesophageal echocardiography	(TOE)
Computed tomography	(CT)
Transmitral pressure gradient	(TMPG)
Intravascular lithotripsy	(IVL)
Percutaneous mitral commissurotomy	(PMC)
